# Treatment of T-Cell Prolymphocytic Leukemia with Central Nervous System Involvement Using Intrathecal Alemtuzumab Administration

**DOI:** 10.1155/2020/8822172

**Published:** 2020-07-27

**Authors:** Jinichi Mori, Kumi Oshima, Satoshi Kimura, Takayuki Ikezoe

**Affiliations:** ^1^Department of Hematology, Jyoban Hospital, Tokiwa Foundation, Fukushima, Japan; ^2^Patient Safety Division, QI Center, St. Luke's International Hospital, Tokyo, Japan; ^3^Department of Hematology, Fukushima Medical University, Fukushima, Japan

## Abstract

T-cell prolymphocytic leukemia (T-PLL) is a rare hematologic cancer with a dismal prognosis. Although a small number of patients have central nervous system (CNS) involvement, a standard treatment approach for these patients has not been established. Herein, we present a case of T-PLL with CNS involvement that was treated with a higher dose of intrathecal alemtuzumab than that previously reported. A 66-year-old man who had T-PLL with CNS involvement was admitted to our hospital. Intravenously administered alemtuzumab, a monoclonal antibody against the CD52 antigen, successfully reduced leukemia cells in peripheral blood; however, intrathecal treatment with methotrexate, cytarabine, and prednisone had a limited effect on the CNS involvement. Therefore, we intrathecally injected alemtuzumab as an experimental treatment. Although we escalated the dose of intrathecal alemtuzumab up to 5 mg, no adverse reaction was noted; however, this treatment did not completely clear the leukemia cells from the patient's cerebrospinal fluid (CSF). We performed whole brain and whole spinal irradiation therapies and subsequently performed allogeneic transplantation from a human leukocyte antigen-matched sibling donor with a conditioning regimen containing total body irradiation. At 21 days after transplantation, leukemia cells remained in his CSF. Although intrathecal alemtuzumab did not eliminate the CNS-invading leukemia cells, it was well-tolerated in our case. Further accumulation of similar cases is needed to determine the benefits and safety of intrathecal alemtuzumab administration.

## 1. Introduction

T-cell prolymphocytic leukemia (T-PLL), a rare hematologic cancer, is associated with poor patient prognosis; the overall 3-year survival rate is 40% after allogeneic hematopoietic stem-cell transplantation (HSCT) in patients who have previously received conventional chemotherapies [1]. Induction therapy with alemtuzumab, a humanized monoclonal antibody against the CD52 antigen, and subsequent allogeneic HSCT may promote longer survival; however, overall, the 4-year survival rate remains at 56% [2]. Few reports have described central nervous system (CNS) involvement, a dismal prognostic factor, in patients with T-PLL [3–5]. For transplantation candidates, pre-HSCT control of CNS involvement is needed to achieve improved transplantation outcomes, as in other types of leukemia [6]; however, a standard treatment approach for cases of T-PLL with CNS involvement does not exist. To date, only one previous report has demonstrated a case where 3 mg of intrathecal alemtuzumab was safely administered; however, the efficacy of this treatment was not evaluated in that case. Here, we present a case of T-PLL with CNS involvement that was treated with up to 5 mg of intrathecal alemtuzumab, but was not eliminated.

## 2. Case Presentation

**Figure 1:**
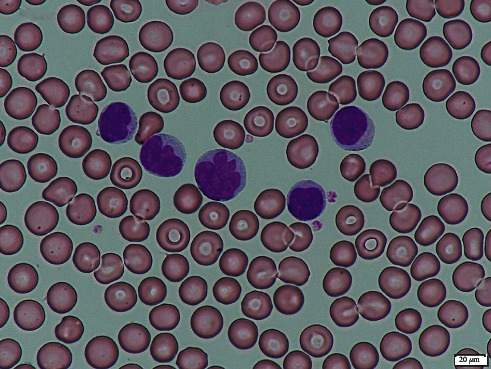
Microscopic image of leukemic lymphocytes in the peripheral blood smear.

A 66-year-old man was diagnosed with T-PLL in 2011. At the time of diagnosis, he was asymptomatic and had a peripheral white blood cell (WBC) count of 17,600 *μ*L, including an abnormal lymphocyte count of 15,000 *μ*L. Thus, his attending physician chose an observant waiting approach. The patient's lymphocyte count gradually increased and reached 30,000–40,000 *μ*L in 2018. He developed night sweats, fatigue, low-grade fever, and headaches. Computed tomography revealed hepatosplenomegaly, and in 2019, he was moved to our hospital to initiate treatment. At the time of the transfer, his WBC count was 52,600 *μ*L, including a lymphocyte count of 46,800 *μ*L. Microscopic examination of the blood smear revealed small-to-medium-sized lymphocytes that featured irregularly shaped nuclei and condensed chromatin (Figure 1).

**Figure 2:**
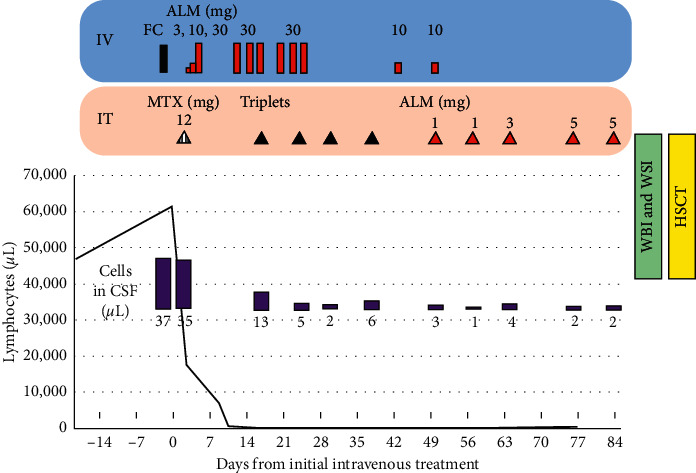
Treatment time course for the leukemia and central nervous system involvement. ALM: alemtuzumab; CSF: cerebrospinal fluid; FC: fludarabine and cyclophosphamide; HSCT: hematopoietic stem-cell transplantation; IT: intrathecal treatment; IV: intravenous treatment; MTX: methotrexate; WBI: whole brain irradiation; and WSI: whole spine irradiation. Triplets consist of 15 mg of methotrexate, 40 mg of cytarabine, and 20 mg of prednisolone.

The lymphocytes exhibited a post-thymic T-cell phenotype (Tdt^−^, CD1^−^, CD5^+^, CD2^+^, and CD7^+^) and harbored chromosomal abnormalities (add(15)(q24) and del(7)(p?)). As the patient was experiencing headaches, we performed a cerebrospinal fluid (CSF) examination and found leukemic lymphocytes carrying the same pattern of surface antigens as those in the peripheral blood. As the initial chemotherapy (fludarabine and cyclophosphamide) caused tumor lysis syndrome, we discontinued the treatment and started administering intravenous alemtuzumab after the resolution of the tumor lysis syndrome. Following these changes, the patient's lymphocyte count dropped sharply (Figure 2).

We concurrently treated the CNS involvement with a dose of intrathecal methotrexate and followed it by intrathecal therapy containing methotrexate, cytarabine, and prednisone weekly. However, the number of CNS-invading leukemic cells reached the lowest point after the fourth intrathecal injection, and the number of leukemic cells in the patient's CSF increased. Once we received approval from our institutional review board, we initiated an experimental treatment with intrathecal alemtuzumab injections. The patient did not experience any adverse reactions to the intrathecal alemtuzumab injections. To eliminate the leukemia cells in the patients' CSF, we increased the intrathecal alemtuzumab dose to 5 mg; however, the cells did not completely disappear. We performed whole brain and whole spinal irradiation therapies and subsequently performed allogeneic transplantation from an HLA-matched sibling donor with a conditioning regimen containing total body irradiation. At 21 days after transplantation, 23 leukemia cells *μ*L remained in his CSF. The patient is currently receiving best supportive care.

## 3. Discussion

We demonstrated here a patient with T-PLL and CNS involvement that was treated with intrathecal alemtuzumab. This treatment unfortunately did not clear the CNS-invading leukemia cells but allowed us to bridge to allogeneic transplantation without any adverse reactions. To the best of our knowledge, this is the first report of a case in which the efficacy and tolerance to intrathecal alemtuzumab injections were evaluated. In a previous report by Alsawah et al., the authors claimed successful treatment with 3 mg of intrathecal alemtuzumab for CNS involvement that was refractory to prior treatment with intrathecal cytarabine and methotrexate and whole brain irradiation [7]. However, when intrathecal alemtuzumab administration was initiated in their case, the patient's CSF leukemic cell count was 1 *μ*L, limiting the evaluation of the treatment efficacy. In contrast, in our case, the patient had a CSF leukemic cell count of 3 *μ*L at the initiation of intrathecal alemtuzumab treatment. Unfortunately, our patient's CNS involvement did not resolve, despite the use of a higher dose (5 mg) of intrathecal alemtuzumab than that previously reported.

Several *in vitro* studies have demonstrated that alemtuzumab exerts its antitumor effect by both complement-dependent cytotoxicity (CDC) and antibody-dependent cellular cytotoxicity (ADCC) [8–10]. Although the effect *in vivo* has not been studied extensively, a preclinical study using human CD52 transgenic mice showed that ADCC plays a crucial role in the elimination of CD52-positive lymphocytes [11]. The study also demonstrated that intravenous administration of alemtuzumab cleared CD52-positive lymphocytes from the peripheral blood but not from certain organs (spleen and thymus) that contain fewer ADCC effector cells, such as neutrophils, natural killer cells, and macrophages. In our patient, intrathecally injected alemtuzumab might have provoked weak CDC and no ADCC in the CSF, which had complement levels that were 100–200-fold lower than those in the serum and extremely few ADCC effector cells. Thus, we speculate that this resulted in the lack of a significant response to the CNS-invading T-PLL cells [12].

Although the efficacy of intrathecal alemtuzumab therapy was limited in our case, it was well-tolerated even at a dose of 5 mg. Further information from similar cases is needed to determine the benefits and safety of this treatment.

## Data Availability

In accordance with the provisions of the ethics committee, personal information about the patient cannot be disclosed.
